# *BRAF* inhibition with concomitant tumor treating fields for a multiply progressive pleomorphic xanthoastrocytoma

**DOI:** 10.2217/cns-2017-0032

**Published:** 2018-04-30

**Authors:** Rimas V Lukas, Ryan T Merrell

**Affiliations:** 1 Department of Neurology, Lurie Cancer Center, Northwestern University, Chicago, IL 60611, USA; 2 Department of Neurology, Northshore University HealthSystem, Evanston, IL 60201, USA

**Keywords:** *BRAF*, Optune, pleomorphic xanthoastrocytoma, tumor treating fields, vemurafenib

## Abstract

Pleomorphic xanthoastrocytomas can be very resistant to treatment if they progress after standard therapy with surgery and radiation. We present the case of a patient with a multiply recurrent pleomorphic xanthoastrocytoma which demonstrated a sustained partial response to a combination regimen of the BRAF inhibitor vemurafenib and tumor treating fields. The regimen proved tolerable and efficacious in this case.

**Summary points**

- Recurrent pleomorphic xanthoastrocytomas (PXA) are often resistant to treatment.
- More than half of PXA habor *BRAF* mutations.
- *BRAF* inhibition with concurrent TTF in our patient with recurrent PXA was well tolerated.
- *BRAF* inhibition with concurrent TTF in our patient with recurrent PXA was associated with radiographic partial response.

Pleomorphic xanthoastrocytomas (PXA) are rare low-grade glial tumors of the central nervous system. Despite their low histologic grade, when not cured via gross total resection they have the potential to continue to recur and/or progress to high grade tumors through all subsequent lines of therapy. Our knowledge regarding optimal management is limited by the lack of prospective trials due to the relative rarity of the disease. As PXA is a rare tumor and prospective trial data does not exist to help guide management, a case report (despite its intrinsic limitations) such as this, still holds value for clinicians treating refractory patients and investigators contemplating potential therapeutic interventions to study.

Over half of PXAs harbor the oncogenic V600E mutation of the *BRAF* gene [1,2]. This mutation is found across numerous cancers and is amenable to treatment with small molecule targeted therapies. The use of tolerable and efficacious monotherapy with a BRAF inhibitor, either vemurafenib [3–5] or dabrafenib [6,7] has been described in recurrent PXA.

Tumor treating fields (TTF) are a novel therapeutic modality targeting intracranial tumors via the use of electrical fields generated by arrays applied directly to the scalp. TTF are US FDA-approved for newly diagnosed [8,9] and progressive glioblastoma [10] but are being investigated in other intracranial tumors such as meningiomas as well as extracranial tumors such as lung and pancreatic cancers. In preclinical models, the efficacy of the TTF was dependent on the frequency of the electrical field and its relationship to the size of the target cell [11,12].

## Case report

The patient was a 23-year-old female who presented in 2010 with new headaches and underwent a surgical resection of a left posterior frontal paramedian PXA (WHO grade 2). When there was recurrence approximately 2 years later she underwent a reresection followed by radiation to 56 Gy. She underwent a third resection when there was rerecurrence approximately 2 years later. The tumor remained grade 2 and was found to harbor the V600E *BRAF* mutation as well as *MGMT* promoter methylation. Craniotomy was followed by nine cycles of temozolomide. Approximately 1 year later, she underwent a fourth craniotomy for progressive disease. At that time, the patient started a regimen of alternative therapies outside of our institutions. Within 6 months, there was further tumor progression. At that time, treatment with a BRAF inhibitor was discussed with the patient. Although the underlying rationale was explained, the patient and family declined due to concerns of side effects. However, she agreed to bevacizumab, and the tumor initially showed radiographic response. However, there was further tumor progression approximately 5 months after starting bevacizumab.

**Figure 1.**
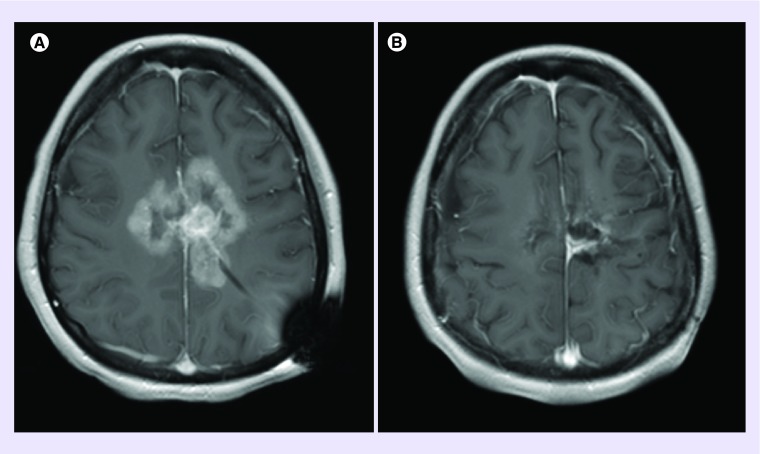
**MRI prior to and following treatment with BRAF inhibitor and concurrent tumor treating field.** Axial T1 post-contrast MRI images from **(A)** 7 February 2017, and **(B)** 8 August 2017.

The patient agreed to treatment with BRAF inhibitor, and vemurafenib was started at a dose of 960 mg every 12 h. Due to the multiply recurrent nature of the tumor, per the patient and family's request, TTF was added to the regimen. The patient achieved a marked partial response with 70% reduction of enhancing tumor after 2 months on combination vemurafenib and TTF. Based on response to vemurafenib and thought that synergism could achieve prolonged efficacy, the MEK inhibitor, trametinib was added. The BRAF and MEK inhibiting combination proved intolerable for the patient, so all treatment was held for 1 month. Then, vemurafenib in conjunction with TTF were resumed. After 2 months on the above-described regimen there was additional partial response on the order of additional 70% reduction of enhancing tumor. The regimen was continued and after 4 months on treatment there was an additional decrease in tumor size with a partial clinical improvement. (Figure 1) Unfortunately, the response was not sustained and the patient died of progressive disease over 37 weeks after initiating the regimen of vemurafenib and TTF.

As the patient was deceased this report was deemed exempt from review by the institutional review board.

## Discussion

PXA while often indolent, have the potential to recur. Recent work in PXA notes a 3 year survival of 80.2% and 5 year survival of 74%. Progression-free survival is 66.5% at 3 years and 60.5% at 5 years [13]. Recurrent PXA can be very refractory to treatment. Although our case may not mimic the natural history of the majority of PXA it is not representative of an esoteric outlier as a substantial percentage of patients will experience progression of disease and death in less than 5 years. Progressive PXA lack adequate treatment options.

BRAF inhibitors have proven beneficial in the management of tumors including *BRAF*-mutated melanomas. TTF have demonstrated efficacy in glioblastoma, a high-grade brain tumor [8–12]. In the case described above advantage was taken of a specific molecular target (V600E *BRAF* mutation) commonly found in these tumors. The use of TTF for PXA has been reported in abstract form [14], but to our knowledge it has not previously been reported in the peer reviewed literature. Due to the lack of known overlapping toxicities the decision was made to add TTF to the regimen. No additional safety signal was detected. However, the addition of a MEK inhibitor to this regimen proved to be intolerable for our patient. The regimen demonstrated notable radiographic improvement in this extremely treatment resistant tumor. Unfortunately, the response was not sustained. Due to the combined nature of the treatment regimen it is impossible to attribute to a specific agent any favorable outcomes. It is also impossible to discern any potential synergy between the two therapeutic modalities. In our case, the combination appeared relatively safe and was associated with sustained partial response of disease.
